# What do dermatologists and dermatology residents think about their residency training in dermatology?

**DOI:** 10.3389/fmed.2023.1293927

**Published:** 2023-12-20

**Authors:** María Librada Porriño-Bustamante, Agustín Buendía-Eisman, Salvador Arias-Santiago

**Affiliations:** ^1^Dermatology Department. University Hospital La Zarzuela, Madrid, Spain; ^2^School of Medicine, University of Granada, Granada, Spain; ^3^Dermatology Department. University Hospital Virgen de las Nieves, Granada, Spain; ^4^School of Medicine, Institute of Biosanitary Investigation ibs, Granada University, Granada, Spain

**Keywords:** education, dermatology, training, residency, survey

## Abstract

**Introduction:**

In Spain, graduates in medicine take a state exam which, depending on their result, enables them choose a speciality in a specific hospital. Becoming a specialist in dermatology involves 4 years of training in a hospital. The content of the speciality is government regulated, although it can vary slightly in different hospitals. Feedback about this training period could provide key information to improve any failings. The aim of the study was to evaluate the perspectives of dermatologists and dermatology residents toward residency training.

**Materials and methods:**

Resident dermatologists in their final 2 years and recently qualified dermatologists answered a survey regarding the residency, and personal perspectives and objectives within dermatology.

**Results:**

A total of 54 participants answered the survey. Their mean age was 29.26 years old. Around 74% of them had had at least 3 clinical sessions per week during their residency and 87% of them considered these clinical sessions to have been useful for their training. The main shortcomings in their training were perceived as laser and esthetics, followed by trichology then research and contact dermatitis. However, 85.2% of them had done external rotations to progress in the areas they felt they needed to improve. Around 55% of the participants had considered specializing in research. Regarding research doctorates, 20.4% were doing their PhD or had already finished it, and of the remaining, 62.79% were interested in doing one.

**Conclusion:**

The opinions, feelings and aspirations of the dermatology residents are an important barometer for the future of the speciality. Training in research, laser and esthetics was perceived as a weakness by the participants, while clinical sessions and external rotations were considered good for their training. The information from this survey establishes a reference point from which present needs and future trends can be gauged.

## Introduction

Dermatology residency training is not standardized worldwide. Each individual country decides how training is conducted and so it varies significantly. In Spain, medical-surgical dermatology and venereology is a regulated speciality in accordance with the Royal Degree (RD) 127/1984 (1). After taking the national MIR (*Médico Interno Residente*) examination, the medical graduates choose their speciality and the hospital depending on their results. Residency training in Dermatology has been in high demand over the past 10 years (2013–2023) and is the first speciality to fill all its places. The training period in Dermatology lasts 4 years and its content is registered in the legislation SCO/2754/2007 (2).

Lifestyle and social media have led to a higher desire for visual perfection which has resulted in an increased demand for the services of dermatologists. This need for cosmetic intervention, along with a “dermatologist drain” to the private medical sector, have led to a lack of dermatologists in the public health system (3).

The future of dermatology resides in our residency training programs. Knowing the opinion of the dermatology residents and newly qualified dermatologists regarding the training period is a key factor in identifying the strengths and also the weaknesses of the system, which is essential in order to improve it. There has only been one other study made to collect the opinions of Spanish dermatology residents regarding their training period (4).

The aim of the study was to evaluate the perspectives of dermatologists and dermatology residents toward residency training. This was carried out by conducting a survey, which included questions about different areas of the training period.

## Materials and methods

The current survey was a cross-sectional study which was conducted online, in Spain, during the month of July, 2023. A 30-question survey was created using Google Forms^®^, and was sent by email to all the participants. The survey was distributed to the target audience by the Spanish Academy of Dermatology and Venereology. Some social networks, specific to dermatology, were also used. The survey was conducted voluntarily and anonymously. The original survey was written in Spanish, although an English version is provided in this article (Annex).

The participants were: (a) Dermatology residents who were going to finish their residency in 2024 (fourth year residents) or 2025 (third year residents); and (b) Dermatologists who had finished their residency between 2019 and 2023, inclusive.

The survey included some demographic data, such as sex, age, year of dermatology residency completion, the hospital chosen for the residency and the number of dermatology residents per year. This survey contained questions to identify the strong and weak points of the residency, to collect information about teaching and researching, as well as questions to clarify current trends among residents and their envisaged career paths and aspirations. Teaching was assessed principally by asking questions about clinical sessions and external rotations, although self-reliance in the management of patients and questions regarding “on-call” days were also addressed. Strengths and weaknesses of the hospital chosen for the residency, in the different areas of teaching relating to the specialty of dermatology, were also questioned. One question referred to the knowledge the participants felt they lacked the most when they started their working lives. Moreover, the participants were asked whether they had intended to work in the public or private systems, or both, when they had started the residency and also when they had finished it. Some questions referred to research during the residency and also about the intention of focusing on scientific research afterwards. Their interest in doing a PhD thesis and teaching were also registered. Some questions about the length of the residency were also included. Certain questions only permitted one answer, but others allowed several answers and even free text. Finally, a SWOT matrix (Weaknesses, Threats, Strengths and Opportunities) was added.

Continuous data are presented as the mean (standard deviation) and categorical data as the relative (absolute) frequency. Qualitative variables were analyzed with the χ^2^ test. Differences were considered significant at *p* ≤ 0.05 and nearly significant at *p* ≤ 0.1. SPSS software (SPSS 20.0, SPSS Inc., Chicago, IL, United States) was used for data analysis.

## Results

**Figure 1:**
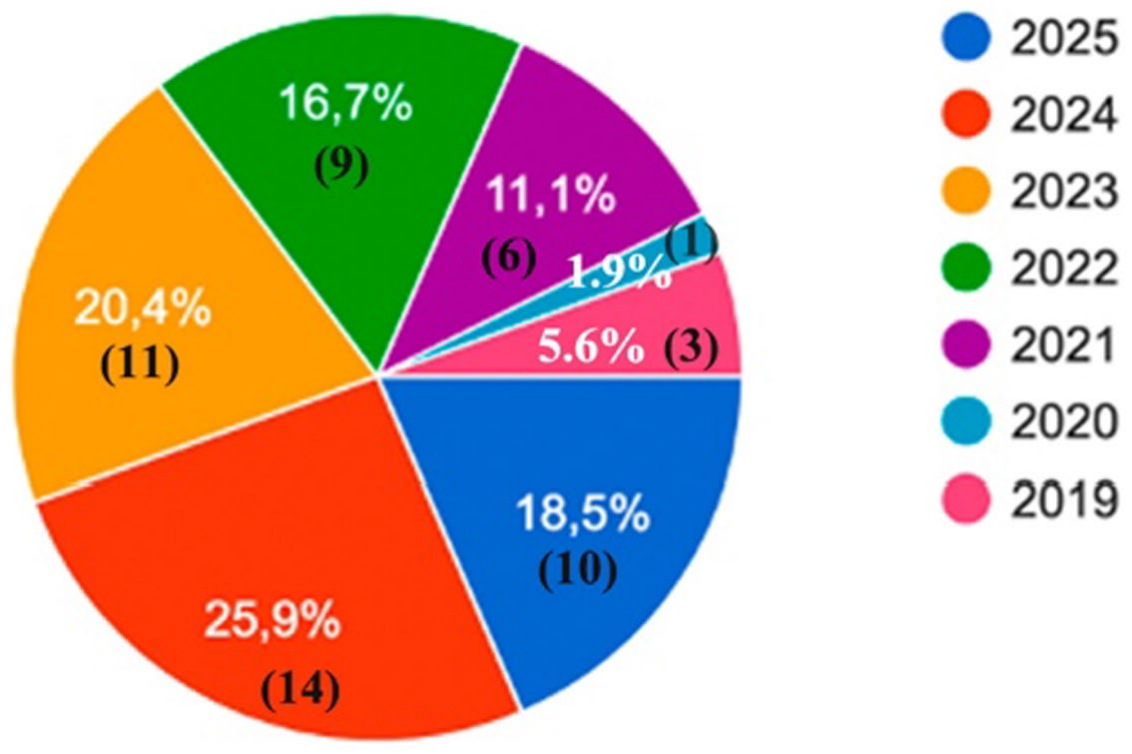
The year in which the participants finished or will finished their residency.

The email was sent to 385 subjects, but it is unknown how many opened it. Finally, a total of 54 participants (30 men, 24 women) responded to the survey. The mean age was 29.26 (SD 2.56), ranging from 26 to 38 years. Almost half of the participants were still residents in the last 2 years of their residency, who were due to finish it during 2024 (25.9%–14/54; fourth year residents) or 2025 (18.5%–10/52; third year residents; Figure 1). Regarding those who had already finished their residency, the higher rate of responses came from those who had just finished it, in 2023 (20.4%–11/54), followed by those who had finished it in 2022 (16.7%–9/44), 2021 (11.1%–6/54), 2019 (5.6%–3/54) and 2020 (1.9%–1/54; Figure 1). Based on the places chosen for dermatology residency, a total of 28 Hospitals were included in this survey (Table 1).

**Table 1:** Answers to the different questions of the survey.

N 54	Answers	Relative (absolute) frequency
Location of the Hospitals in charge of the training of the participants	Madrid	37% (20)
	Andalusia	16.7% (9)
	Valencian Community	13% (7)
	Catalonia	11.1% (6)
	Basque Country	7.4% (4)
	Castilla-León	5.6% (3)
	Castilla-La Mancha	5.6% (3)
	Murcia	3.7% (2)
Residents per year	One	27.8% (15)
	Two	46.3% (25)
	Three	13% (7)
	Four	13% (7)
Frequency of clinical sessions	Several per week (3–5)	74.1% (40)
	One per week	22.2% (12)
	One every two weeks	1.9% (1)
	One per month	1.9% (1)
	Less than one per month	0
Usefulness of the clinical sessions	I think they are indispensable	87% (47)
	I think they are good, but dispensable	13% (7)
Optimal frequency for clinical sessions	Several per week (3–5)	74.1% (40)
	One per week	20.4% (11)
	One every two weeks	1.9% (1)
	One per month	3.7% (2)
	Less than one per month	0
Encouragement to study during the residency	Yes, it encouraged me to study	59.3% (32)
	No, it did not encourage me to study, but I did it anyway	33.3% (18)
	No, I did not study	7.4% (4)
Independent management of patients	From the 1^st^ year	20.4% (11)
	From the 2^nd^ year	42.6% (23)
	From the 3^rd^ year	11.1% (6)
	From the 4^th^ year	20.4% (11)
	Never	5.6% (3)
On-call days	Yes, complete working day	29.6% (16)
	Yes, but half working day	48.1% (26)
	No, never	22.2% (12)
Usefulness of the on-call days in residency (in the case of answering Yes in the previous question)	They are useful to improve our training plus the extra-salary we received for them	92.86% (39/42)
	They are useful only because of the extra-salary	2.38% (1/42)
	They are useless	2.38% (1/42)
	No response	0.02% (1)
Possibility for doing external rotations	Yes	85.2% (46)
	No	14.8% (8)
Usefulness of the external rotations	I did some and they were really useful	83.33% (45)
	I think they are useful, but I have not been allowed to do them or I have not been able to do them	14.81% (8)
	I did some and they were not really useful to me	1.9% (1)
	I did not do any rotation, but I think they would not have been really useful to me	0
Preference for public or private practice, or both, at the beginning of the residency	Public practice	22.2% (12)
	Private practice	9.3% (5)
	Public + private practice	68.5% (37)
Preference for public or private practice, or both, at the end of the residency	Public practice	13% (7)
	Private practice	9.3% (5)
	Public + private practice	77.8% (42)
Participation in research projects and interest in doing it	I participated in research projects and I expect to continue doing it	70.4% (38)
	I participated in research projects but I do not consider to continue doing it	9.3% (5)
	I did not participate in research projects, but I will consider doing it in the future	18.5% (10)
	I did not participate in research projects, and I do not expect to do it in the future	1.9% (1)
Interest in specializing in research in dermatology	It is my priority and I am going to do it	3.7% (2)
	I am going to do it, but it is not my priority	51.9% (28)
	I have not considered it	9.3% (5)
	I would not like to do it, but I could consider it if I found a good opportunity	29.6% (16)
	None	5.6% (3)
Writing or reviewing scientific articles during the residency	I did it, and I expect to continue doing it	70.4% (38)
	I did it, but I do not expect to continue doing it	18.5% (10)
	I did not do it, but I expected to do it	7.4% (4)
	I did not do it and I do not expect to do it in the future.	3.7% (2)
PhD thesis in the residency or currently	I have already finished my PhD thesis	1.9% (1)
	I am currently working on my PhD thesis	18.5% (10)
	I have not done any PhD thesis	79.6% (43)
Interest in doing a PhD thesis (in the case of answering the third option in the previous question)	Yes	62.79% (23/43)
	No	37.21% (16/43)
Interest in teaching in dermatology	Yes, I am already teaching at the University	18.5% (10)
	Yes, but I do not teach at the University yet	72.2% (39)
	None	9.3% (5)
Usefulness of a final test after the residency to assess the acquired skills	Yes, an OSCE-type test (Objective Structured Clinical Examination – ECOE in Spanish) and a theoretical test would be appropriate	20.4% (11)
	Yes, an OSCE-type test would be appropriate	7.4% (4)
	Yes, a theoretical test would be appropriate	7.4% (4)
	Not necessary	64.8% (35)
The need to increase the residency by one year	Yes	20.4% (11)
	No	79.6% (43)
The need to do sub-specializations in the residency (increasing its duration)	Yes	37% (20)
	No	63% (34)

**Figure 2:**
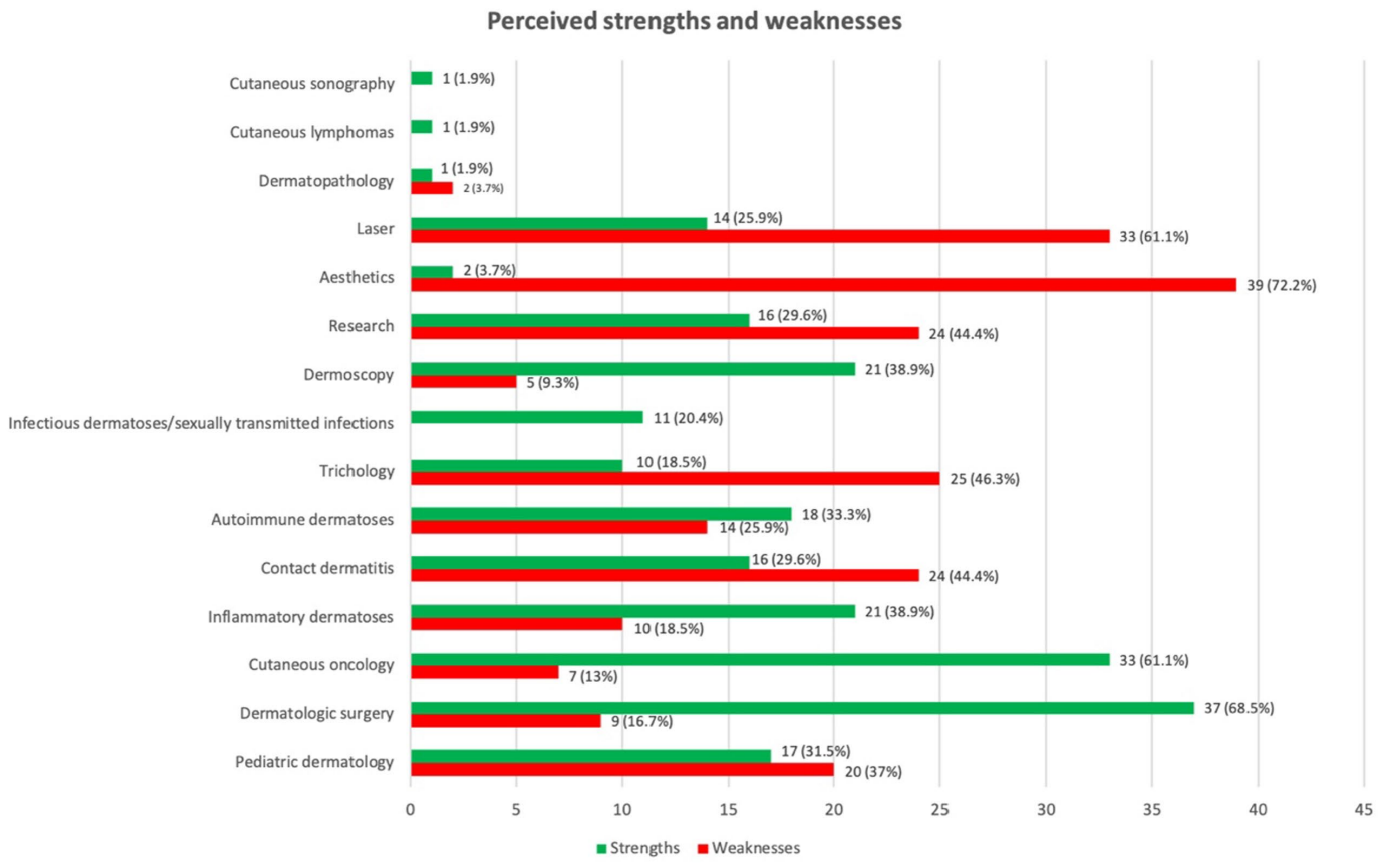
Perceived strengths and weaknesses in the training content.

During the residency, the areas of the speciality of dermatology perceived as strengths and weaknesses by the participants are represented in the Figure 2. Most of the participants (85.2%) mentioned being able to do specific external rotations to improve the weak areas of their training. The most popular areas for these rotations were: pediatric dermatology, sexually transmitted diseases, dermatopathology, cutaneous oncology and dermatologic surgery (including micrographic Mohs surgery) and laser, followed by trichology, esthetics, cutaneous ulcers, skin sonography, bullous and autoimmune diseases and contact dermatitis. The time spent on these rotations usually ranged from 1 to 5 months, although one participant spent 7 months on them. The longest rotations were usually a combination of rotations in oncology and dermatologic surgery, laser and esthetics, pediatric dermatology and dermatopathology.

**Figure 3:**
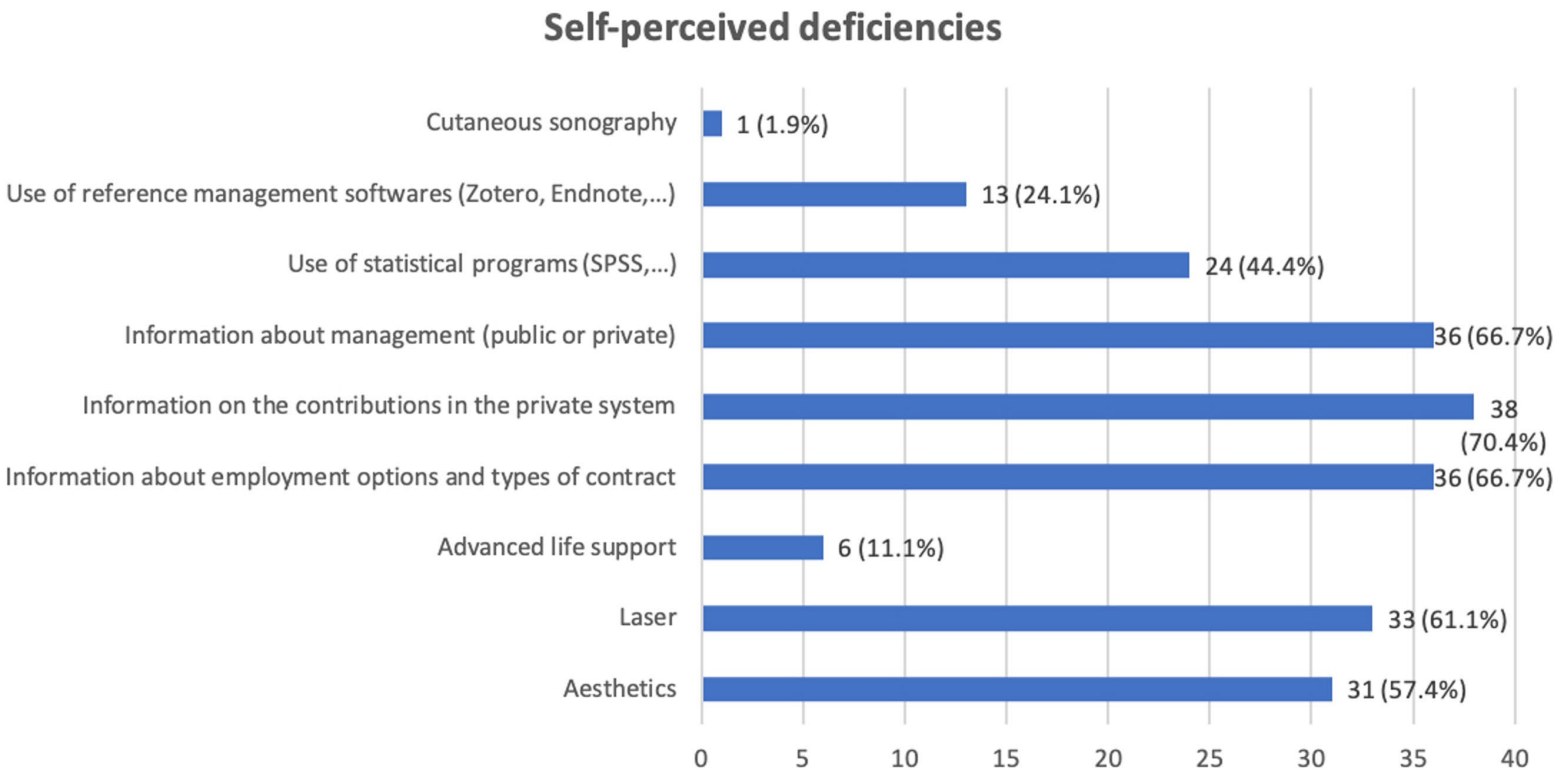
Self-perceived shortfalls when facing their professional life.

With regards to the external courses performed during the residency, the perceived shortcomings when facing working life are represented in the Figure 3.

With regards to the PhD thesis, 11 out of 54 patients (20.37%) started their PhD thesis during the residency period, and one of them had already finished it.

**Figure 4:**
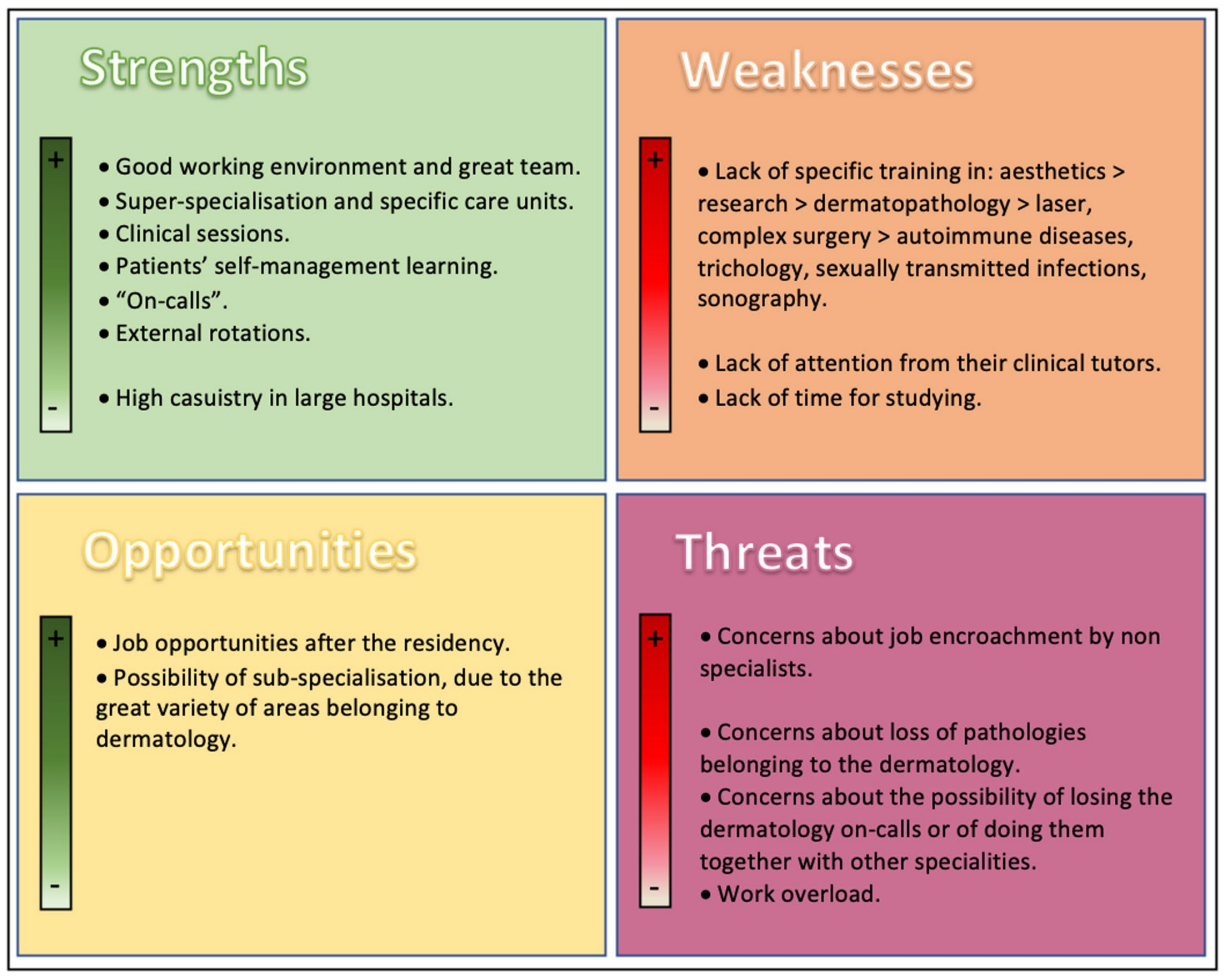
SWOT matrix with the most common responses of the participants.

Regarding the SWOT matrix, the main responses (the ones repeated by a higher number of participants) are presented in Figure 4. Other weaknesses commented on by some of the participants were: the existence of differences between hospitals regarding the possibility of doing external rotations and “on-call” days, the lack of innovation, the low casuistry in small hospitals, the impact on residents when working with professionals who had suffered or were suffering burnout in the public system, the lack of encouragement for the PhD thesis, the lack of supervision from specialists when “on-call,” the lack of checks for the study by the tutors, the high number of residents per year (hospital with 4 residents/year) and the dermatology service’s dependency on the residents, along with the lack of respect for the external rotations already established because of “service needs” (resident-dependent services). With regards to SWOT Threats, the most commonly referred to by the participants was, by far, the concerns about job encroachment by non-specialists. Other SWOT Threats commented on by the participants were the dehumanization of patient care due to the lack of time, the lack of regulation of specific objectives in the training period and the lack of time for studying due to an excessive number of courses. Regarding the SWOT Strengths, the most commonly referred to were the good working environment and great teams, and the possibility of super-specialization and working in specific care units. Most of the participants agreed with the two SWOT Opportunities cited in Figure 4. Other opportunities referred to by some participants were: the possibility of doing external rotations in private centers, the possibility of working in the private sector when having finished the residency, and the existence of a great variety of courses and research scholarships.

No significant differences were noted between any of the variables assessed and the sex of the participants.

## Discussion

This study is the result of a survey conducted in Spain about education in the residency of dermatology. It was answered by 54 participants, who were residents of dermatology in their last 2 years of training or already dermatologists in their first 5 years of practice.

Only one report about the dermatology residency in Spain had been previously published by Montero-Vílchez et al. (4). It included 52 residents in their third year of training and analyzed different issues about the training of the future dermatologists. They found that a mean of 3 clinical sessions were organized per week in each department, somewhat consistent with our results, since 74.1% of the participants referred to having at least 3 clinical sessions per week and considered it the best frequency for them. In Spain, clinical sessions are usually half or a third of the day sessions, at the beginning or the end of the working day, while the remaining time is normally dedicated to patient-care tasks. Clinical sessions were considered good for their training by all of the participants, and in fact, indispensable by 87% of them. This result was also consistent with the data found by a Canadian study, in which 81% of the dermatology residents surveyed ranked clinical sessions as a very important component in training (5).

The lowest score in the level of residency satisfaction, also found in the previous Spanish study, was for training in research (4), which was also perceived as a weakness by 44.4% of the participants of the present study and one of the weaknesses referred to by them in the SWOT matrix. On the other hand, the highest score they found in the level of satisfaction was the one on external rotations (4), which was one of the strengths written down in the SWOT matrix. Around 85% of the participants in our survey did external rotations in order to make up for the shortcomings in their training. Moreover, the external rotations were considered to be useful by all but one of the participants who did them and even by those who were not able to do any.

Regarding the interest of working in the public or private system, five of those who at the beginning of the residency wanted only public practice changed their mind in favor of public plus private practice when they finished their residency. So that, 77.8% of the participants decided to work in both systems at the end of the residency, similar to the 75% found in the previously published Spanish article (4). However, in that study, none of the residents wanted to work exclusively in private health care, while in ours, 9.3% wanted to do it at the beginning of their residency and also at the end of it.

In the matter of cosmetic training, laser and esthetics were both considered weaknesses in the training by our participants (61.1 and 72.2% of them, respectively) and they also were perceived as deficiencies when facing their working life. This seems to be common in other countries too, since a survey among dermatology residents in Jordan showed that 78.6% of them were unsatisfied with their training in cosmetic dermatology (6). Moreover, some studies about Canadian and United States residents also reported dissatisfaction with cosmetic dermatology training (5, 7, 8). However, despite this, they seem to receive more specific training about cosmetics during their residency than the Spanish residents. A recent study found that in the United States almost every program provides hands-on cosmetic dermatology training during dermatology residency (botulin toxin, skin fillers, skin peels and lasers) (8). As the demand for cosmetic dermatology is increasing, an appropriate training is necessary, probably starting during the residency. Luckily, most of the participants referred to having the option of doing external rotations to fill in some of the gaps they felt existed in their training.

On the subject of research, more than 50% of the Canadian residents stated that the research component should be mandatory in the residency (5). Interestingly, 88.89% of our participants were interested in doing research in their professional life, and 55.6% had even considered a specialization in research. However, more than 40% of our participants felt that research was a weakness in their training. The residents of the Montero-Vílchez et al.’s study considered that patient-care tasks largely made teaching and research more difficult, which was also a complaint of our participants. The main barriers the participants referred to having, for doing research, were the lack of time, followed by the lack of support from their tutors and the lack of encouragement.

In the present survey it was found that 20.37% of the participants started their PhD thesis during the residency period, and one of them had already finished it. This is a higher percentage than the 11.5% found in the afore mentioned Spanish study (although they only considered third year residents) (4). Considering the aspirations of the new dermatologists, research may be a training area which should be improved in the residency and also the time available for doing it. The Canadian study found that significantly more men than women indicated an interest in academics, research and teaching (5). Nevertheless, in the current survey, no differences regarding sex were found regarding the interest in doing a PhD thesis, researching and teaching.

On one hand, most of the participants surveyed did “on-calls” during their residency (77.7%) and found them useful for their training (92.86%). Moreover, some of the participants considered the possibility of having the “on-calls” removed or absorbed by other specialties as a threat in the SWOT matrix. In fact, the “on-calls” may be a very appropriate option for improving the independent management of patients.

On the other hand, the SWOT matrix revealed that the participants wished for closer monitoring of their theoretical and practical training, similar to the results found in the previous Spanish article (4). As the SWOT matrix reflected, the participants lacked sufficient support from their tutors, similar to the Canadian residents, who expressed their desire for more teaching and mentorship (5). A United States survey about mentoring in dermatology residents found an association between increased mentoring and training satisfaction (7). However, as happens in education, good teaching is often neither fostered nor appropriately rewarded (5). Teaching and mentorship should be valued as highly as publication and research.

In the United States, at the time of completion of residency, residents currently take the American Board of Dermatology’s primary certification examination, and passing this is required to become a board certified in dermatology (9). Currently, in Spain there is no examination after finishing the residency and most of the participants surveyed (64.8%) considered that a final test is not necessary.

Regarding other self-perceived shortfalls after finishing the residency, in addition to the training in esthetics and laser previously referred to, most of the participants felt they lacked essential information about working life, management, employment options, types of contract and social contributions. This content is not included in the training program of the dermatologists, but it is relevant information that every physician should know, so, some teaching about working issues may be advisable during the residency.

There are some limitations in this study. First, the small sample size of the cohort, and of some subgroups, limits the power of inferential statistics to detect clinically relevant response differences. However, the analysis of subgroups was not the aim of this study, and the survey itself can already provide some interesting data for improving the training of dermatology residents. Another limitation is the lack of responses among dermatologists who finished their residency more than 2 years ago, although in these group recall biases could be more common because of the time passed.

To conclude, this article shows the current vision of the young dermatologists and final year residents surveyed about their residency, expressing self-perceived shortcomings and strengths, their interest in teaching or research, and the handicaps they found when starting their independent working life. The improvement of these factors could strengthen the speciality by upgrading the education of future generations of dermatologists.

## Data availability statement

The original contributions presented in the study are included in the article/Supplementary materials, further inquiries can be directed to the corresponding author.

## Author contributions

MP-B: Conceptualization, Data curation, Formal analysis, Investigation, Methodology, Writing – original draft, Writing – review & editing. AB-E: Conceptualization, Project administration, Supervision, Writing – review & editing. SA-S: Conceptualization, Methodology, Project administration, Supervision, Writing – review & editing.
