# Supplementary material for: What do dermatologists and dermatology residents think about their residency training in dermatology?
Source: Front Med (Lausanne). 2023 Dec 20;10:1293927. doi: 10.3389/fmed.2023.1293927 (PMC10773702; doi:10.3389/fmed.2023.1293927)
Supplement: Supplementary file 1 [file Data_Sheet_1.docx]

**Annex:** Survey about Education in dermatology residency

1. Age: ___

2. Sex: Male/Female

3. Year of finishing the residency: 2025 / 2024 / 2023 / 2022 / 2021 / 2020 / 2019

4. Hospital chosen for the residency: ___

5. Number of residents per year in the hospital of the residency: one / two / three / four

6. Number of clinical sessions during service:

- Several per week (3-5).

- One per week.

- One every two weeks.

- One per month.

- Less than one per month.

7. Do you feel that clinical sessions were/are helpful for your training?

- Yes, they are indispensable.

- Yes, they help me, but are dispensable.

- No, they are not really helpful.

8. Which one do you consider is the best frequency for clinical sessions during the residency?

- Several per week (3-5).

- One per week.

- One every two weeks.

- One per month.

- Less than one per month.

9. Do you consider that in the residency there is an adequate encouragement for further study?

- Yes, study is/was encouraged.

- No, study is/was not encouraged, but I study/studied anyway.

- No, I do/did not study so much.

10. Regarding independent management of patients, when did you have/have you got this possibility?

- Since the first year of residency.

- Since the second year of residency.

- Since the third year of residency.

- Only since the fourth year of residency.

- Never.

11. Did/do you do “on-calls” in the residency?

- Yes, a complete working day.

- Yes, but half a working day.

- No, I do/did not do “on-calls”.

12. If you have answered yes in the question number 11, do you think the “on-calls” are necessary during the residency?

- Yes, they helped/help me to improve my training plus the extra salary I received/receive for them is welcome.

- Yes, but only because of the extra salary I received/receive for them.

- No, I think they are not necessary and I would prefer not to have done them/not to do them, even if it means having a lower salary.

13. Which ones do you consider the strong points in your training in your hospital? You can choose various options.

- Pediatric dermatology.

- Dermatologic surgery.

- Cutaneous oncology.

- Inflammatory diseases.

- Contact dermatitis.

- Autoimmune diseases.

- Trichology.

- Infectious diseases / sexually transmitted infections.

- Dermoscopy.

- Research.

- Aesthetics.

- Laser.

- (Free answer)

14. Which ones do you consider the weak points in your training in your hospital? You can choose various options.

- Pediatric dermatology.

- Dermatologic surgery.

- Cutaneous oncology.

- Inflammatory diseases.

- Contact dermatitis.

- Autoimmune diseases.

- Trichology.

- Dermoscopy.

- Research.

- Aesthetics.

- Laser.

- (Free answer)

15. Could you do specific external rotations to improve what you considered the weak points in your training as a dermatologist?

- Yes, I could.

- No, I could not.

16. If you have answered yes in the question number 15, what external rotations did you do and how long did they take? ______

17. Do you think that external rotations are helpful during the residency?

- Yes, I did some and they seemed very helpful to me.

- Yes, but I could not do them.

- No, I did some and they did not seem very helpful to me.

- No, I did not do any, and I think they are not helpful.

18. When you started the residency, what kind of practice did you think that you would choose after finishing the residency?

- Only public practice.

- Public + private practice.

- Only private practice.

19. When you finished the residency (or now, close to the end), what kind of practice do you think that you will choose after finishing the residency or what have you chosen?

- Only public practice.

- Public + private practice.

- Only private practice.

20. Several courses are offered during the residency to complete the training, are you lacking in any area when it comes to facing your working life?

- Aesthetics.

- Laser.

- Advanced life support

- Information about employment options and types of contract.

- Information on the contributions in the private system.

- Information about management (public or private).

- Use of statistical programs (SPSS,…).

- Use of reference management softwares (Zotero, Endnote,…).

- (Free answer)

21. Did you participate/have you participated in research projects during the residency or are you considering doing it in your professional practice?

- I participated/have participated in research projects during the residency and I expect to continue doing it.

- I participated/have participated in research projects during the residency but I do not expect to continue doing it.

- I did not participate/have not participated in research projects during the residency but I would consider doing it.

- I did not participate/have not participated in research projects during the residency and I am not considering to do it.

22. Would you like to specialise in research in dermatology?

- It is my priority and I am going to do it.

- I am going to do it, but it is not my priority.

- I have not considered it.

- I would not like it, but I could consider it if I found a good opportunity.

- None.

23. Did you write or review/have you written or reviewed scientific articles during your residency? Do you expect to do it in your professional practice?

- I did it, and I expect to continue doing it.

- I did it, but I do not expect to continue doing it.

- I did not do it, but I would consider doing it.

- I did not do it and I would not consider doing it.

24. Did you start/have you started a PhD thesis during your residency?

- Yes, I have already finished my PhD thesis.

- Yes, I am currently working on my PhD thesis.

- No, I have not done any PhD thesis.

25. If you have answered No in question number 24, would you like to do a PhD?

- Yes, I would.

- No, I would not.

26. Are you interested in teaching in Dermatology?

- Yes, I am already teaching at the University.

- Yes, but I do not teach at the University yet.

- No, I am not.

27. Do you think it would be helpful to do a test at the end of the residency to assess the skills acquired?

- Yes, an OSCE-type test (Objective Structured Clinical Examination – ECOE in Spanish) and a theoretical test would be appropriate.

- Yes, an OSCE-type test would be appropriate.

- Yes, a theoretical test would be appropriate.

- No, I do not think it is necessary.

28. Do you think it is necessary to increase the residency period by one year?

- Yes, I do.

- No, I do not.

29. Do you think it is convenient to do sub-specialisations during the residency (increasing its duration)?

- Yes, I do.

- No, I do not.

30. SWOT matrix (free answers).
